# Benzophenone-3 does not Cause Oxidative Stress or B-esterase Inhibition During Embryo Development of *Octopus maya* (Voss and Solís Ramírez, 1966)

**DOI:** 10.1007/s00128-023-03788-4

**Published:** 2023-10-31

**Authors:** Gissela Moreno-Ortiz, Letícia Aguilar, Claudia Caamal-Monsreal, Elsa Noreña-Barroso, Carlos Rosas, Gabriela Rodríguez-Fuentes

**Affiliations:** 1 Posgrado en Ciencias del Mar y Limnología, Universidad Nacional Autónoma de México, Av. Universidad 3000, Ciudad Universitaria, Coyoacán, Ciudad de México, C.P. 04510 México; 2 Unidad de Química en Sisal, Facultad de Química, Universidad Nacional Autónoma de México, Puerto de Abrigo s/n, Sisal, Yucatán México; 3 Unidad Disciplinaria de Docencia e investigación, Facultad de Ciencias, Universidad Nacional Autónoma de México, Puerto de Abrigo s/n, Sisal, Yucatán México

**Keywords:** Benzophenone-3, Antioxidant system, Oxidative stress, *Octopus maya*

## Abstract

Benzophenone-3 (BP-3) is an active ingredient in sunscreen lotions and personal-care products that protects against the damaging effects of ultraviolet rays. Given its worldwide dissemination, it has been linked with harmful effects on aquatic biota; however, its impact is not fully understood calling for further studies. To understand the impacts on an important economically and ecologically species, we evaluated the toxicity of BP-3 during the embryonic development of *Octopus maya.* Embryos were exposed to increasing concentrations of up to 500 µg BP-3/L until hatching. Antioxidant enzyme activities, oxidative-stress indicators, and B-esterases activities were measured at different developmental phases (organogenesis, activation, and growth). There were no significant differences between treatments, suggesting the lack of production of toxic metabolites that may be related to a protective chorion, an underdeveloped detoxification system, and the experimental conditions that limited phototoxicity.

## Introduction

Knowledge of the harmful effects of ultraviolet radiation on the skin like inflammation, photo-aging, sunburn, and cancer, led to increased use of ultraviolet (UV) filters. UV filters are a group of chemicals that absorb or reflect UV irradiation. Among the most used UV filter is benzophenone-3 (BP-3; 2-hydroxy-4-methoxy benzophenone; oxybenzone) (Wang et al. 2011). BP-3 is an organic UVB and UVA filter. It has been widely employed in many personal care products as a UV protectant(Chisvert et al. 2012) and in plastics as a UV stabilizer (MacVicar et al. 2022).

BP-3 has been reported in 95% of wastewater effluents and 86% of surface waters globally (see Scheele et al. 2023). It has been detected in marine environments across the world at concentrations ranging from 3.7 ng/L to 27,880 ng/L (Tovar-Sánchez et al. 2013; Tashiro and Kameda 2013; Tsui et al. 2014; Sánchez Rodríguez et al. 2015; Downs et al. 2022; Pei et al. 2023). BP-3 is lipophilic and may potentially bioaccumulate. Its K_ow_ value (3.6) suggests slow biodegradation, a tendency to adsorb to suspended solids and sediments, and low volatilization potential from the water surface (Kim and Choi 2014). Those physicochemical properties indicate its persistence in aquatic environments, linked to negative impacts on biota (Matouskova and Vandenberg 2022).

Data from different species reveal contradictory results, depending on the tested concentrations, with most studies reporting effects on freshwater species (Carvalhais et al. 2021). Most of the information in marine environments is related to negative impacts on corals. Reports of coral reef bleaching and mortality through oxidative-stress-mediated processes show its adverse effects under light or dark conditions (Danovaro et al. 2008; Downs et al. 2016). In the marine flatfish (*Scophthalmus maximus*), there were no significative differences in metabolic profile, oxidative stress, or neurotoxicity after three and seven days of intraperitoneal injection of BP-3 (3 µg per g fish weight) (Carvalhais et al. 2021). However, there is not much information on other marine species.

Cephalopods have a wide diversity and distribution. They are an important ecological and economic resource. It has been reported that they could be susceptible to chemical pollution (Rodrigo and Costa 2017). Most toxicological studies with cephalopods are related to the accumulation of trace elements (Penicaud et al. 2017) and polycyclic aromatic hydrocarbons (Aguilar et al. 2023). However, the physiological and molecular mechanisms leading to negative responses and detoxification must be better understood (Rodrigo and Costa 2017).

The Mexican four-eyed octopus, *Octopus maya*, is an endemic species of the Yucatan continental shelf. This species sustains one of the most important cephalopod fisheries in Mexico, where 80% of the catch is exported to Europe and Asia(Rosas et al. 2014) and whose catch is constantly doubling (Santamaría et al. 2023). Females of *O. maya* spawn an average of 28 +/- 13 clusters, with 20–30 eggs each, with an average production of 680 +/- 100 eggs/female (Rosas et al. 2014). At 24 °C, embryonic development takes place in a period of between 45 and 55 days.

*O. maya* possess large eggs with an average of 15 mm in length (Rosas et al. 2014), and are encapsulated in a chorionic membrane or chorion which assumes the function of protecting the embryo against unfavorable environmental factors (Monsalvo-Spencer et al. 2013). This species could be exposed to contamination by BP-3 because the tourists and residents of the region intensively use sunscreens. In support of this statement, the discharge of UV filters into marine environments increased from 137 tons in 2003 to 217 tons in 2019 (Casas-Beltrán et al. 2021).

The main objective of this study was to understand the toxic effects of BP-3 during the embryo development of *O. maya*. It is well known that chemicals can alter the balance between prooxidant and antioxidant defense (Regoli and Giuliani 2014); we evaluated the antioxidant enzymes activities (catalase, CAT; superoxide dismutase, SOD; glutathione-S-transferase, GST), total glutathione concentration (GSH) and indicators of oxidative damage (protein carbonylation, PO; and lipid peroxidation, LPO). B-esterases have also been used as important biomarkers of exposure and effect. Traditionally they have been used in environmental monitoring to measure the exposure of the organisms to organophosphate and carbamate pesticides present in the aquatic environment (Sanchez-Hernandez 2007). Still, it has been demonstrated that many other chemicals are B-esterase inhibitors (Solé et al. 2022) and that some esterases are inhibited by oxidative stress (Schallreuter et al. 2004; Rico et al. 2007).

### Methodology

The experimental design was carried out after our protocol was approved by the experimental Animal Ethics Committee of the Faculty of Chemistry at Universidad Nacional Autónoma de México with permit number FQ/CICUAL/460/22.

Rosas et al. (2014) and Sanchez-García et al. (2017) previously described the captivity and maintenance of reproductive organisms. After spawning, thirty-six clutches of eggs were used for the experiment. The experimental design was carried out with six treatments, two controls (control and solvent control with DMSO 0.01%), and 5, 25, 50, and 500 µg/L of BP-3 (Sigma-Aldrich ≥ 98%). Each experimental unit consisted of acrylic tanks with three chambers connected with a seawater recirculation system to provide a suitable environment for embryonic development (Rosas et al. 2014). Each treatment was tested in triplicate.

The seawater used was treated with biological and ultraviolet (UV) filtration. Seawater temperature was controlled to 24 ± 2 °C, a salinity of 36 PSU, 7 mg/L of dissolved oxygen, and light intensity 5 lx cm^− 2^ with a photoperiod of 12:12 h light- darkness. To ensure the welfare of eggs, 50% of seawater was changed every three days. Samples of water (10 mL) were collected from each replicate and for each treatment to determine the real BP-3 concentration by solid-phase microextraction, and gas chromatography-mass spectrometry (SPME/CG) as previously described by Rodríguez-Fuentes et al. (2015).

Egg samples were taken every five days after exposition during embryo development, 4 eggs per tank. A total of 401 eggs were sampled and analyzed. In each sampling, embryos were photographed using Leica EZ4 HD (Wetzlar, DE) stereoscopic microscope whose software (Leica LAS EZ, Wetzlar, DE) allows further identification of the embryonic stage. The development stages were identified according to Deryckere et al. (2020) and Naef (1928) and were separated into three critical phases: organogenesis (before hearts activity started; stages X to XIV), activation (when the branchial and systemic hearts began their activities; stages XV to XVI) and growth (stages XVII to XX to the time of hatching). Total exposure of embryos lasted forty days.

Individual eggs were snap-frozen in liquid nitrogen and stored at -80 °C until further analysis. Samples were homogenized with 0.05 M tris Buffer pH 7.4 at 1:50 (v/w), using a Potter Elvehjem homogenizer with a PTFE pistil immersed in ice. PO, LPO, and GSH are reported per mg of wet tissue. The rest of the homogenate was centrifuged at 10,000 rpm for 5 min at 4° C (Eppendorf Centrifuge 5424 R, United States), and the supernatant was separated for analysis. Activities were calculated per mg of protein.

AChE activity was determined byEllman et al. (1961) method adapted to a microplate by Rodríguez-Fuentes et al. (2008). The reaction started by adding acetylcholine iodide (1 mM) and was measured at an absorbance of 405 nm for 120 s for 2 min. CbE activity was determined using the method by Hosokawa and Satoh (2002); the reaction started by adding Tris Buffer 7.4/ƿNPA solution, and the reaction was measured at an absorbance of 405 nm every 15 s for 5 min. CAT activity was determined using the molybdate method byGóth (1991), modified by Hadwan and Abed (2016), by measuring the reduction rate of hydrogen peroxide at 405 nm every 15 s for 10 min upon reaction with ammonium molybdate. GST levels were obtained based on the Sigma Aldrich Assay Kit CS04 following the method byHabig & Jakoby (1981) with CDNB 100 mM as the substrate for spectrophotometric measurement at 340 nm every 15 s for 5 min. SOD activity was determined by Sigma Aldrich Assay Kit 19,160. Total GSH concentration was determined by Sigma Aldrich Glutathione Assay Kit CS0260. PO quantification was based on the method by Mesquita et al. (2014), and LPO quantification was carried out by the FOX method using Sigma Aldrich PeroxiDetect Kit. Proteins were analyzed in the supernatant following Bradford (1976), using bovine serum albumin (BSA) as a standard to standardize all enzyme activities in activity unit (U) mg ^− 1^ protein.

The data were analyzed using a multivariate approach. Permutational MANOVA (PERMANOVA) and Principal Coordinate Analysis (PCO) were performed using Primer v 7.0 + PERMANOVA add-on. Data were transformed using the function log10(x + 1) and normalized. The resemblance was calculated using the Euclidean Distance of samples (Legendre 2019). PERMANOVA was done using the permutation of the residuals under a reduced model with 9999 permutations to generate pseudo-F (Anderson 2001). Permutational multiple pair-wise tests were used to compare the centroids of the combination of two factors, the development phase, and treatments.

## Results and Discussion

In marine environments, octopuses are under environmental and pollution stress that could affect the resilience of these organisms. In this work, we evaluated the effect of BP-3 in *O. maya* during embryo development. We exposed eggs to control (seawater), control solvent (seawater with DMSO, 0.01%), and 5, 25, 50, and 500 µg/L of BP-3 in DMSO 0.01%. The two first concentrations could be found in marine environments (Downs et al. 2022), while the others were used to exacerbate the effects. During the exposure of embryos, water samples were taken every three days from each replicate and for each treatment (n = 132). The mean (± standard deviation) measured concentrations of BP-3 were 3.66 ± 0.86, 19.72 ± 2.77, 30.57 ± 4.90, 298.65 ± 74.39 µg/L, respectively, for nominal concentrations of 5, 25, 50, and 500 µg/L. BP-3 was below the limit of quantification (1 µg/L) in the control and control solvent treatments.

**Fig. 1:**
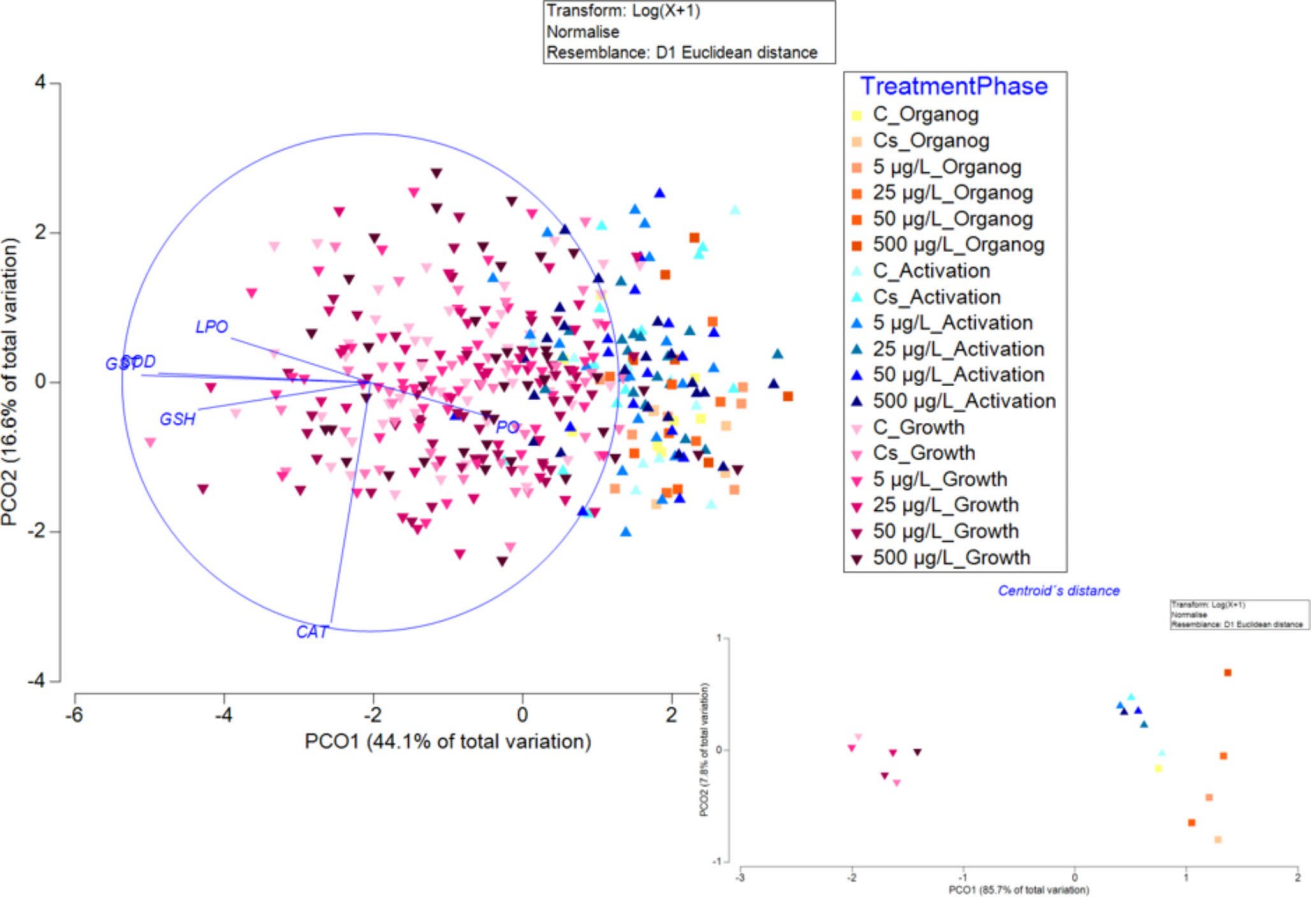
Principal coordinate analysis (PCO) of antioxidant enzymes and oxidative biomarkers, AChE and CbE enzyme activities in embryos of *O. maya* exposed to Control (C), Control solvent (Cs), 5, 25, 50 and 500 µg/L of BP-3 at organogenesis, activation and growth phases. A 2-D representation of the relative distance and location of within-group centroids is embedded

The PCO applied to antioxidant enzymes and damage indicators of *O. maya* eggs during embryo development exposed to different treatments of BP-3 explained 60.7% of the total variation in the first two principal coordinates (Fig. 1). The eigenvectors that contributed most to the sample separation in the first coordinate (horizontal) were GST (-0.92258), SOD (-0.85354), and to a lesser extent, GSH (-0.69434), PO (0.49508), and LPO (-0.56141), and show the antioxidant enzymes activities increased while embryos developed. For the second principal coordinate, CAT (-0.96509) was the unique eigenvector that contributed. The PERMANOVA showed no significant interaction between the phase of embryo development and treatments (pseudo-F = 0.806; p ˃ 0.05; 9855 unique permutations), and there was no effect of BP-3 exposure concentration (pseudo-F = 0.5258; p ˃ 0.05; 9899 unique permutations). However, there was a significant difference between phases (pseudo-F = 56.297; p = 0.0001; 9936 unique permutations), where eggs at the growth phase presented the highest antioxidant enzymatic activities and total GSH concentration, which can be observed in the centroids (Fig. 1). During *O. maya* embryo development, embryos undergo different phases. It is possible to observe how the antioxidant system is activated, from very low activities and GSH concentration at the organogenesis phase to high values at the growth phase. Similarly, recent studies have shown that from the end of organogenesis to the growth phase there is a significant increase in the enzymatic and nonenzymatic components of the antioxidant system in this species (Domínguez-Castanedo et al. 2023).

Our obtained results suggest that no observable effects could be related to an underdeveloped detoxification system, where the enzymes are not yet fully active in embryos, as has been reported byBlüthgen et al. (2012) in embryos of *Danio rerio*. They found that in eleuthero embryos only BP-3 was detected in all samples, in contrast with adult fish, where BP-3 and BP-1 (2,4-dihydroxybenzophenone) were found. Previous studies have shown that BP-1 is the main metabolite produced by BP-3 via hydroxylation (Kim and Choi 2014). BP-1 has an extra -OH group, which might increase the toxic potential of this metabolite (Mao et al. 2022). However, the mechanism of biotransformation in cephalopods still needs to be fully understood.

BP-3 is considered relatively stable under UV light and artificial sunlight (Kim and Choi 2014), because of the two benzene rings and the carbonyl group present in its molecule which offered photoprotection without photodegradation or inducing phototoxicity even after a prolonged time of irradiation by UV-light (Abid et al. 2017). Although, previous studies suggest that BP-3 is phototoxic, exacerbating effects under UV radiation generated the production of hydroxyl radical (•OH), significantly increasing the toxicity (Zhang et al. 2021). However, there are few BP-3 studies under UV conditions. For example, in zebrafish embryos, the presence of UV light significantly potentiated the toxicity of BP-3 generating a 96 h-LC50 of 2.30 mg/L in contrast to the UV light with a 96 h-LC50 of 4.74 mg/L (Zhang et al. 2021). In a study with the sea symbiotic Anemone *Aiptasia* under UV conditions, BP-3 (2 mg/L) was metabolized into phototoxic glucoside conjugates, which cause the death of organisms, whereas BP-3 in the absence of UV cause little to no mortality (Vuckovic et al. 2022). Since our study was done at 1–5 Luxes to simulate the environmental conditions of octopus embryo development, it is unlikely that photooxidation will occur. However, it is necessary to consider this factor in older organisms exposed in natural environments.

In aquatic animals, embryonic development is a period of exponential cell expansion (Siefert et al. 2015). Thereby, embryos are surrounded by the chorion, a special biological structure that ensures stability inside the egg (Boletzky 2003). The chorion protects embryos against unfavorable environmental factors such as desiccation, bacterial infection, and physical destruction and could prevent many pollutants from entering (Duan et al. 2020). The protective effect of chorion against pollutants has been identified by the enhancement of sensitivity to exogenous compounds after chorion removal in zebrafish embryos (Yang et al. 2019; Vranic et al. 2019). However, in cephalopods, the chorion has not yet been classified according to the characteristics of its structures, nor its biochemical composition (Monsalvo-Spencer et al. 2013) and/or if the chorion has chemical or electrochemical properties to avoid chemicals.

**Fig. 2:**
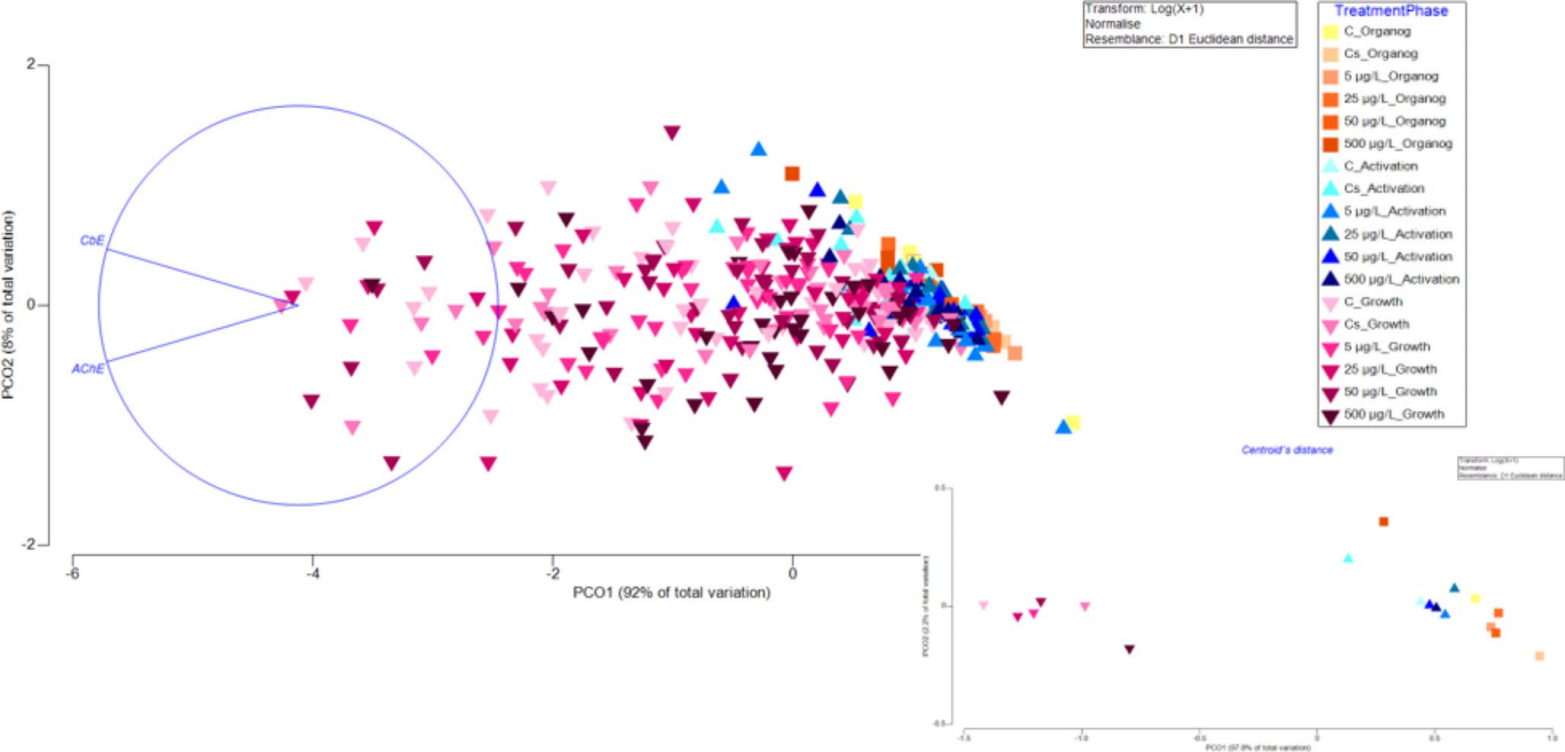
Principal coordinate analysis (PCO) of AChE and CbE enzyme activities in embryos of *O. maya* exposed to Control (C), Control solvent (Cs), 5, 25, 50 and 500 µg/L of BP-3 at organogenesis, activation, and growth phase. A 2-D representation of the relative distance and location of within-group centroids is embedded

Additionally, our results showed that BP-3 did not affect AChE and CbE activities. PERMANOVA showed no significant interaction between the phase of embryo development and treatments (pseudo-F = 0.7642; p ˃ 0.05; 9930 unique permutations), there was no effect of BP-3 concentration (pseudo-F = 0.10652; p ˃ 0.05; 9943 unique permutations). The graph of centroids in Fig. 2 shows that activities increased during embryo development (pseudo-F = 85.142; p = 0.0001; 9952 unique permutations), AChE activities increased as the embryo grows, as has been previously described in *Octopus sp* (Sanchez-García et al. 2017; Olivares et al. 2019). AChE is expressed in several tissues, including the nervous system and muscle and its activity is essential during early development(Behra et al. 2002). CbE plays an essential role in embryonic growth and development via fat metabolism (Lian et al. 2018). The present study showed no evidence of B-esterase inhibition at different BP-3 concentrations, as described in *D. rerio* (Sandoval-Gío et al. 2021).

In conclusion, we explore the effects of BP-3 during *O. maya* embryo development. The results showed that BP-3 does not cause oxidative stress or B-esterase inhibition during this life stage. These findings may be related to physiological mechanisms that give them adaptive capabilities to cope with stressors (Somero 2010; Pazzaglia et al. 2021; de Paula et al. 2022), for example, an underdeveloped detoxification system in the embryo stage that reduce the production of toxic metabolites despite of the presence of a protective chorion during most of the exposure, and the experimental conditions that reduce the production of phototoxic by-products. It is necessary to continue the study of phase I, phase II reactions, and BP-3 metabolites in this species throughout their life cycle to understand better the mechanisms involved in UV filters’ toxicokinetic and toxicodynamic.
